# Is There a More Practical Way for Screening Minimal Hepatic Encephalopathy?

**DOI:** 10.5152/tjg.2023.23197

**Published:** 2023-06-01

**Authors:** Şerife Değirmencioğlu Tosun, Coşkun Özer Demirtaş

**Affiliations:** 1 Bezmi Alem University Faculty of Medicine Hospital, İstanbul, Turkey; 2 Division of Gastroenterology and Hepatology, Marmara University Faculty of Medicine, İstanbul, Turkey

Dear Editor,

We read with great interest the recently published original article by Eyice et al^1^ where they documented the role of critical flicker frequency (CFF) test and psychometric tests (PST) in the detection of minimal hepatic encephalopathy (MHE). Accordingly, the prevalence of MHE in patients with chronic liver disease was found to be 13% and 14% using the CFF and PST, respectively. The combined use of CFF and PST has shown that it increases the recognition rate of MHE by up to 24% in these patients. The importance of detecting MHE has been well described considering its results including decreased attention, memory problems, or decreased learning capacity which can cause catastrophic consequences.^2,3^ However, the diagnosis of MHE is generally complicated by the lack of convenient and reliable tools suitable for use in daily practice.

The CFF test has advantages in the evaluation of attention abnormalities. It can be easily understood and applied after 10-minute training. But the test is not specialized in assessing motor function, suggesting that the test could only detect certain types of HES. It was mentioned that the CFF test is superior in patients with alcohol-related liver cirrhosis and may cause inaccurate measurements in those who are colorblind and have vision problems. Instruments used for CFF testing may affect test results if misplaced on the face.^4^ The PST is required to be carried out in the presence of an expert and in appropriate physical conditions. Considering these facts, the PST is difficult to repeat and prone to mistakes, while CFF has the disadvantage of being not easily accessible.

In recent years, smartphone-based applications established as screening tools for the diagnosis of MHE have been proposed to be used as a stepwise algorithm or even as a stand-alone screening tool, such as EncephalApp_Stroop Test which has been externally validated several times.^5^ These applications are easily repeatable and accessible via smartphones without the need for going to the hospital or a physician. The results of these tests can be followed easily by clinicians over the Internet. Patients and their relatives can make their self-checks for MHE from their homes, and the results of these tests can be followed easily by clinicians over the Internet. In this respect, it seems that these smartphone-based MHE tests can make more sense for clinical practice by providing a simple, easy-to-access, and valid dynamic tool for screening MHE.
