# ERRATUM

**DOI:** 10.5152/tjg.2023.230627

**Published:** 2023-07-01

In the Letter to the Editor by Değirmencioğlu Tosun and Demirtaş, entitled “Is There a More Practical Way for Screening Minimal Hepatic Encephalopathy?” (Turk J Gastroenterol. 2023;34(6):672. DOI: 10.5152/tjg.2023.23197) which was published in the June 2023 issue of the Turkish Journal of Gastroenterology, it has come to our attention that an oversight occurred in accurately attributing the affiliation of the second author, Coşkun Özer Demirtaş, within the article. The correct affiliation of the author has been implemented on the online version of this article. You can access the corrected version of the article through the link below:

https://turkjgastroenterol.org/en/is-there-a-more-practical-way-for-screening-minimal-hepatic-encephalopathy-137075

